# Prenatal diagnosis of 17q12 microdeletion and microduplication syndrome in fetuses with congenital renal abnormalities

**DOI:** 10.1186/s13039-019-0431-7

**Published:** 2019-05-17

**Authors:** Shanning Wan, Yunyun Zheng, Yinghui Dang, Tingting Song, Biliang Chen, Jianfang Zhang

**Affiliations:** Department of Obstetrics and Gynecology, The First Affiliated Hospital Of AFMU (Air Force Medical University), 127 ChangLe West Road, Xi’an, 710032 Shaanxi China

**Keywords:** Prenatal diagnosis, Congenital renal abnormalities, Karyotype, Chromosomal microarray-based analysis

## Abstract

**Background:**

Copy number variations (CNVs) involving the 17q12 region are associated with a broad range of clinical phenotypes. Deletion of the 17q12 chromosome results in structural or functional abnormalities in the kidney and urethra, type 5 diabetes (MODY5), and neurodevelopmental or neuropsychiatric disorders. Microduplication of 17q12 is rare and is associated with an increased risk of epilepsy and mental retardation. We studied the prenatal diagnosis of 17q12 microduplication and microdeletion syndrome in fetuses with congenital renal abnormalities.

**Case presentation:**

We conducted a retrospective analysis of prenatal diagnoses in our hospital from January 2016 to April 2018. Abnormal renal ultrasound findings were present in 126 fetuses and the incidence of chromosomal abnormalities was 10.32%(13/126). Conventional karyotyping detected 7 of 126 fetuses as aneuploid (5.56%). In addition, chromosome microarray analysis (CMA) detected 6 fetuses(4.76%) with copy number variations (CNVs), of which 5 were shown to have 17q12 microdeletion syndrome and 1 had 17q12 microduplication syndrome. We followed up these pregnant women. The results of the testing had a significant impact on pregnancy outcome. The phenotypes of 17q12 microdeletions and microduplications vary widely, affecting patients in different ways, such as language delays, social deficiencies, and even abortion.

**Conclusions:**

The characteristics of 17q12 microdeletions and microduplications are so vague that the condition is often misdiagnosed or missed. This study demonstrated that karyotype analysis combined with CMA can significantly improve the diagnostic rate in prenatal diagnosis of CNVs, which can provide evidence for genetic counseling in such pregnancies.

## Background

Congenital anomalies of the kidney occur in 3–7 per 1000 births [1–3]. The prevalence of unilateral and bilateral dysplastic kidneys in the general population is 1 per 1000 and 1 per 5000, respectively [4]. Approximately 10% of patients with anomalies of the kidney have a family history, indicating a genetic pathogenesis underlying these disorders [2–4].

The 17q12 microdeletion syndrome (OMIM 614527) is caused by a deletion of the 17q12 region of the chromosome. The clinical features include renal cysts, diabetes of the young type 5(MODY5) [5–7], Mullerian aplasia, dysgenesis [8, 9], autism spectrum disorder schizophrenia [10, 11], learning difficulties, speech delay, neonatal cholestasis, and transient neonatal hypercalcemia [12]. The main clinical manifestations of chromosome 17q12 microduplication syndrome (OMIM 614526) are developmental delay, brain dysplasia, epilepsy, cognitive impairment, and behavioral abnormalities [13]. Less common phenotypes include esophageal atresia, eye abnormalities, cleft palate, heart defects, and sex reversal [14].

For prenatal diagnosis, traditional karyotyping is still considered the gold standard, however, studies have indicated that for fetuses with abnormal ultrasonographic finding, chromosome microarray analysis (CMA) detection has a significant advantage over karyotyping and has a higher detection rate for chromosomal abnormalities [15]. We present 5 fetuses diagnosed with 17q12 microdeletion syndrome and 1 fetus with 17q12 microduplication syndrome by CMA. Traditional karyotyping combined with the CMA technique was used for prenatal diagnosis of a large number of fetuses with renal ultrasound abnormalities, providing evidence for genetic counseling of renal abnormalities.

## Case presentation

During the period between January 2016 and April 2018, a total of 2161 pregnant women underwent prenatal diagnostic testing in the prenatal diagnostic center of our hospital. Abnormal renal ultrasound findings were presented in 126 fetuses, including hydronephrosis, multi-cystic kidneys, increased echogenicity of the kidneys, and bilateral dysplastic kidneys. The average gestational age at the time of amniocentesis was 23 ± 3 weeks (range, 18–32 weeks).

Traditional karyotyping and CMA were performed in the 126 prenatal samples. Furthermore, parents received genetic counseling, signed informed consent, and had follow-up evaluations until birth or termination of the pregnancy.

## Methods

### Karyotype analysis

Amniotic fluid (20 ml) was sampled at 18–32 weeks gestation by amniocentesis. Giemsa banding was performed (450–550 band resolution), karyotyped, and nomenclature was used according to the 2006 ISCN [16].

### CMA analysis

Genomic DNA from amniotic fluid (10 ml) was extracted using a QIAamp DNA Blood Mini Kit (Qiagen, Venlo, The Netherlands). Analysis of the concentration and quality of genomic DNA were performed using a Nanodrop 2000 (Thermo Fisher Scientific, Waltham, MA, USA).

A CytoScan 750 K array (Affymetrix, Inc., Santa Clara, CA, USA) was used for CMA for each fetus. The experimental procedure was carried out in strict accordance with the manufacturer’s standard protocols (Affymetrix, Inc.), including DNA digestion, ligation, polymerase chain reaction (PCR), fragmentation, labeling and hybridization. The results were analyzed using Chromosome Analysis Suite software (Affymetrix, Inc.). To analyze the data and interpret the results, the following public databases were used: UCSC (http://genome.ucsc.edu); DECIPHER (http://decipher.sanger.ac.uk/); DGV (http://www.ncbi.nlm.nih.gov/dbvar/); OMIM (http://www.ncbi.nlm.nih.gov/omim); ISCA(https://www.iscaconsortium.org/); and PubMed (http://www.ncbi.nlm.nih.gov/pubmed/). According to the American College of Medical Genetics (ACMG) guidelines [17], the copy number variation (CNV) was classified into the following categories: benign, likely benign, variants of unknown significance (VOUS), likely pathogenic, and pathogenic.

### Parental verification

Prenatal samples were assessed by karyotype and CMA according to standard procedures. When the CMA results showed fetal chromosomal abnormalities, the chromosomes of both parents were tested to verify whether the abnormality was de novo or inherited. After informed consent was signed, the peripheral blood of the parents was used for CMA verification.

## Results

**Fig. 1:**
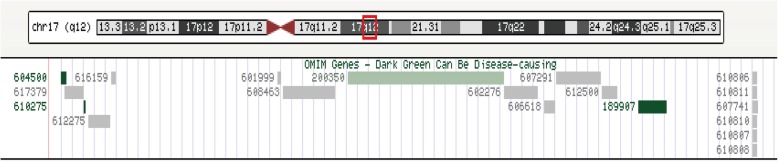
Microarray profile of chromosome 17 showing the deleted region and the corresponding UCSC and OMIM genes. OMIM: Online Mendelian Inheritance in Man; UCSC: University of California Santa Cruz

Among the 126 pregnancies with renal abnormalities detected by ultrasonography, 10.32%(13/126) of the fetuses had chromosomal abnormalities. Conventional karyotyping showed that 7 of 126 fetuses (5.56%) were aneuploid, including trisomy 13 (*n* = 1), trisomy 18 (*n* = 1), and trisomy 21 (*n* = 5). The gravidas all chose to terminate the pregnancies. Among the remaining 119 cases, CMA detected 6 fetuses (5.04%) with pathogenic CNVs, of which 5 fetuses had 17q12 microdeletion syndrome (4.20%), 1 had 17q12 microduplication syndrome (0.84%) and all 6 fetal karyotypes were normal. The identified CNVs were located at chromosome 17q12, which spanned a minimum size of 1.42 Mb and a maximum size of 1.58 Mb (Table 1). According to the public databases, the CNVs were pathogenic, which encompassed 19 OMIM genes, including *HNF1B, LHX1, ACACA*, and others (Fig. 1).The dark green genes can represent disease. Parental CMA results showed that all six CNVs in the fetuses were *de novo*. Two parents eventually chose to terminate the pregnancies and four parents decided to continue the pregnancies (Table 1).

**Table 1:** Pathogenic CNVs of 6 fetuses detected by CMA

*No.*	*Indication*	*CMA result*	*Size (Mb)*	*Pregnancy outcome*
*1*	Multi-cystic kidney	*arr [hg19] 17q12(34,822,465-36,404,555)×1*	*1.58*	*Birth. Polycystic renal dysplasia*
*2*	Separation of renal pelvis	*arr [hg19] 17q12(34,822,465-36,307,773)×1*	*1.48*	*Birth. No obvious abnormality*
*3*	Increased echogenicity of the kidneys	*arr [hg19] 17q12(34,822,465-36,404,555)×1*	*1.58*	*Birth. Abdominal distension*
*4*	Much amniotic fluid	arr [hg19] 17q12(34,822,465_36,404,104)×3	*1.58*	*Birth. No obvious abnormality*
*5*	Hydronephrosis	*arr [hg19] 17q12(34,822,465-36,243,365)×1*	*1.42*	*TOP*
*6*	Bilateral dysplastic kidneys	*arr [hg19] 17q12(34,822,465-36,404,104)×1*	*1.58*	*TOP*

*TOP* termination of pregnancy

We followed-up these families. After the number 1 fetus was born, the follow-up ultrasound revealed polycystic renal dysplasia, and no other abnormalities were observed. At 1 year of age, the child had slow development, poor constitutional features, susceptibility to colds, and was often hospitalized. When the child was 2 years old, the child’s constitution features were better, growth was stunted, expression of language was poor, and the child could not walk. The child has been undergoing rehabilitation training. The mother is pregnant for the second time. Fortunately, the ultrasound and CMA results were normal. When the number 2 and 4 children were born, the results of the newborn examinations were normal. The number 2 child is now 7 months old. He can turn over and sit, and there are no obvious abnormalities. The number 4 child is 4 months old, and the parents reported that the child has normal growth and development and the feeding habits are good. The clinical manifestation of the number 3 child after birth was abdominal distension, a poor digestive system, and difficulty in feeding. The baby was hospitalized in the neonatal department after birth, but the child eventually died. The parents who chose to terminate the pregnancy were diagnosed with apparent fetal defects during the examination in late pregnancy.

## Discussion and conclusion

Our results showed that the combination of karyotyping and CMA technology significantly improved diagnostic rates. Among fetuses that underwent CMA testing, chromosome 17q12 microdeletions and microduplication syndrome were not detected by conventional karyotyping in 5.04% (6/119) of the fetuses. CMA demonstrated a high clinical value and could be the first choice for diagnosis in such pregnancies.

Microdeletion and microduplication of chromosome 17q12 have shown considerable variability in clinical manifestations. Previous studies have shown that in the second trimester, a 17q12 microdeletion of fetuses, including *HNF1B* and *LHX1,* may result in bilateral dysplastic and multi-cystic kidneys [18, 19]. Li et al. [20] reported a 17q12 microduplication in a fetus, and the ultrasound findings showed mild ventricular enlargement, corpus callosum dysplasia, and microcephaly. Rosenfeld et al. [21] found that the penetrance for 17q12 microdeletions and microduplications in the post-natal population was only 34.4 and 21.1%, respectively. Bierhals et al. [22] reported a child with developmental delay, epilepsy, microcephaly, low tension, and dullness who inherited a 17q12 1.4-Mb microduplication from his healthy father. Chen et al. [23] reported a fetus with hydronephrosis, ureteral hydrops, and polycystic kidneys whose chromosome 17q12 1.75-Mb microdeletion was inherited from an unaffected mother.

Our data are consistent with the literature. The results of these 6 fetuses all included two pathogenic genes, but the phenotypes of patients after birth varied widely. The number 1 child had renal dysplasia after birth, accompanied by developmental delay, and a social disorder. The number 2 and 4 children showed no obvious abnormalities. The number 3 child was more severely affected with abdominal distension, difficulty feeding, and eventually died. The number 5 and 6 families eventually chose to terminate the pregnancies because the genitourinary system had significant structural abnormalities in each fetus during the ultrasound examination in the second trimester. The incomplete penetrance of this microdeletion and microduplication syndrome poses a challenge to prenatal counseling. Therefore, in genetic counseling, prospective parents should be informed about the range of results for penetrance and possible phenotypes. If the prospective parents intend to continue the pregnancy, then all the tests during pregnancy must be done to determine whether or not the fetus is normal. In the absence of significant phenotypic abnormalities, neuropsychiatric assessment and monitoring should be guaranteed during childhood and adulthood so that problems can be addressed earlier.

In summary, our results explored the prenatal clinical phenotype of the 17q12 microduplication and microdeletion syndrome. Our results indicated that if conventional karyotyping is combined with CMA testing, the diagnostic rate of chromosomal abnormalities is significantly increased and will have an impact on pregnancy outcomes. The results of this study can provide a basis for obstetricians to provide genetic counseling, improve genetic counseling results, and help many prospective parents.

## Abbreviations

CMA: Chromosomal microarray analysis
CNV: Copy number variation
PCR: Polymerase chain reaction
VOUS: Variation of uncertain clinical significance
